# An uncommon manifestation of COVID-19 pneumonia on CT scan with small cavities in the lungs: A case report

**DOI:** 10.1097/MD.0000000000021240

**Published:** 2020-07-10

**Authors:** Jinqing Chen, Song Peng, Bangjun Zhang, Zhifeng Liu, Lang Liu, Wendy Zhang

**Affiliations:** a Department of Radiology, Xiaochang First People's Hospital, 1 Station Front Road, Xiaochang, Hubei; b Department of Radiology, Chongqing Health Center for Women and Children, 120 Longshan Road, Chongqing, China; c New York Medical College, Valhalla, NY.

**Keywords:** cavity, chest CT, COVID-19 pneumonia, imaging features, laboratory examination

## Abstract

**Rationale:**

Chest computed tomography (CT) scans play a key role in diagnosing and managing of COVID-19 pneumonia. The typical manifestations of COVID-19 pneumonia on a chest CT scan are ground glass opacities, consolidation, nodules, and linear opacities. It can be accompanied by a “crazy-paving” pattern, air bronchograms, pleural hypertrophy, and pleural effusion. However, no literature has reported a case with cavities in the lungs.

**Patient concerns:**

A 34-year-old male patient complained of fever, cough, fatigue, myalgia, diarrhea, headache, and dizziness for 2 weeks. This patient is living in Xiaogan, a city around Wuhan, and he had contact with a patient with COVID-19 pneumonia from Wuhan <14 days before he had fever.

**Diagnosis:**

A nucleic acid test by rRT-PCR returned positive on a pharyngeal swab, confirming the diagnosis of COVID-19 pneumonia.

**Interventions:**

Isolation antiviral treatment.

**Outcomes:**

After 19 days of isolation and antiviral treatment, his temperature returned to normal and the symptoms were relieved. The laboratory results also were returning to normal levels. The chest CT scan showed that the acute inflammation had subsided significantly. With 2 consecutive novel coronavirus nucleic acid tests had returned negative, the patient was discharged from the hospital and sent to a government designated hotel for quarantine observation. The unique chest CT manifestation in this case was the small cavities in both lungs during the absorption phase of this disease. These small cavities developed into consolidated nodules with clear edges and gradually shrank or disappeared.

**Lessons:**

Although 2 consecutive nucleic acid tests returned negative in this patient, the small cavity changes in the lungs were observed, so the patient was quarantined for 14 days. However, follow-up CT after the first 14 days’ quarantine showed new small cavity changes on the lungs, a further 14 days of quarantine was recommended. Therefore, in some COVID-19 cases, even if the nucleic acid tests turns negative, the disappearance of lung lesions may take a long time. The repeated chest CT scan plays an important role in the diagnosis and evaluation of the recovery of COVID-19.

## 1 Introduction

Since December 2019, many cases of viral pneumonia have been detected in Wuhan, Hubei Province of China. On January 12, 2020, the World Health Organization (WHO) named the virus as 2019 novel coronavirus (2019-nCoV).^[1]^ On February 11, 2020, the WHO officially named the disease caused by the novel coronavirus as COVID-19 (Corona Virus Disease-19).^[2]^

Chest computed tomography (CT) scans play a key role in the diagnosis of COVID-19 pneumonia. Its diagnostic value lies in the detection of lesions, the judgment of the nature of lesions, and the assessment of the severity of the disease, so as to facilitate the clinical classification. A previous study showed that the patient had an epidemiological history, and CT scans showed typical COVID-19 pneumonia lesions in the lungs. However, COVID-19 nucleic acid tests by real-time reverse transcriptase polymerase chain reaction (rRT-PCR) returned negative results several times before the final diagnosis was made.^[3]^ Therefore, a CT scan on the chest is very important for the early diagnosis of COVID-19 pneumonia. The typical manifestations of COVID-19 pneumonia at the early stage on a CT scan include multiple small patchy shadows and interstitial inflammation, predominantly distributed in the peripheral third of the lungs. Later, it develops into multiple ground glass opacities and infiltrates in the lungs. Furthermore, pulmonary consolidation was observed, but pleural effusion was rare.^[4]^ From initial diagnosis to patient recovery, CT scans showed significant morphological changes in the lesions, but no literature has reported small cavities in the lungs on a chest CTs as a sign of COVID-19. We present the case of a 34-year-old patient with COVID-19 pneumonia who had typical manifestations of the disease on a CT scan along with constantly changing small cavities in the lungs.

## 2 Case report

**Figure 1:**
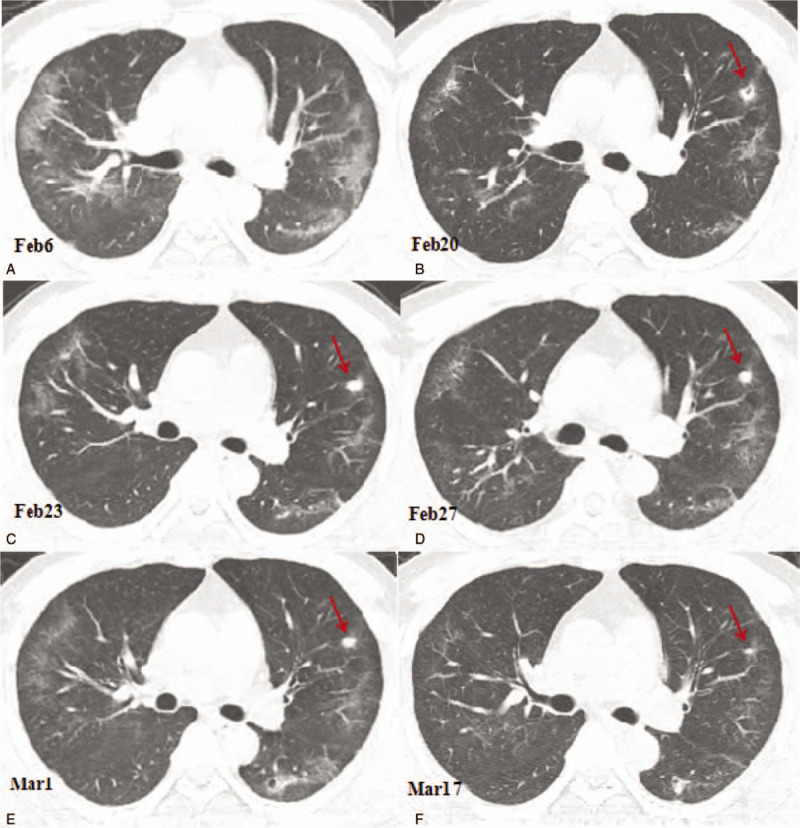
Computed tomography (CT) images showing the changes of the cavity in the anterior segment of the left upper lobe. (A) A CT image obtained on February 6, 2020 showing the ground glass opacities and linear opacities in the lungs predominantly distributed in the peripheral third of the lungs. (B) A CT image obtained on February 20, 2020 showing the reduced ground glass opacities area. A small cavity was found in the anterior segment of the left upper lobe with a size of 9.3 × 6.7 mm. (C) A CT image obtained on February 23, 2020 showing consolidation of the small cavity in the anterior segment of the left lobe with a size of 7.8 × 6.3 mm (arrow); (D) A CT image obtained on February 27 showing consolidation of the cavity in the upper segment of the left upper lobe with the size slightly reduced to 6.5 × 5.5 mm (arrow); (E) A CT image obtained on March 1, 2020 showing the reduced cavity in the anterior segment of the left upper lobe, with a size of 5.0 × 4.5 mm (arrow). (F) A CT image obtained on March 17, 2020 showing that the ground glass opacities in the lungs were almost completely absorbed. The solid nodule in the anterior segment of the left upper lobe had significantly reduced in size, with a size of 2.5 × 1.5 mm (arrow).

A 34-year-old male patient presented to our hospital on February 6, 2020. He complained of fever, cough, fatigue, myalgia, diarrhea, headache, and dizziness for 2 weeks, with a maximum temperature of 39.1°C. He had no hypertension, diabetes, coronary heart disease, and tuberculosis. This patient is living in Xiaogan, a city around Wuhan, and he had contact with a patient with COVID-19 pneumonia from Wuhan <14 days before he had fever. The first CT scan showed multiple ground glass opacities and linear opacities distributed in the peripheral third of the lungs, with no significant lymphadenopathies in the mediastinum and the hilum of the lungs, which was consistent with the typical manifestations of COVID-19 pneumonia (Fig. 1A). A laboratory examination showed that the white blood cell count was 8.57 × 10^9^ cells/L (normal value: 3.5–9.5 × 10^9^ cells/L), the lymphocyte count was 1.89 × 10^9^ cells/L (normal value:1.1–3.2 × 10^9^ cells/L), the C-reactive protein (CRP) level was 44.86 U/mL(normal value: 0–8 U/mL), and the erythrocyte sedimentation rate (ESR) was 67 mm/h (normal value: 0–20 mm/h). On February 11, 2020, a nucleic acid test by rRT-PCR returned positive on a pharyngeal swab, confirming the diagnosis of COVID-19 pneumonia.

**Figure 2:**
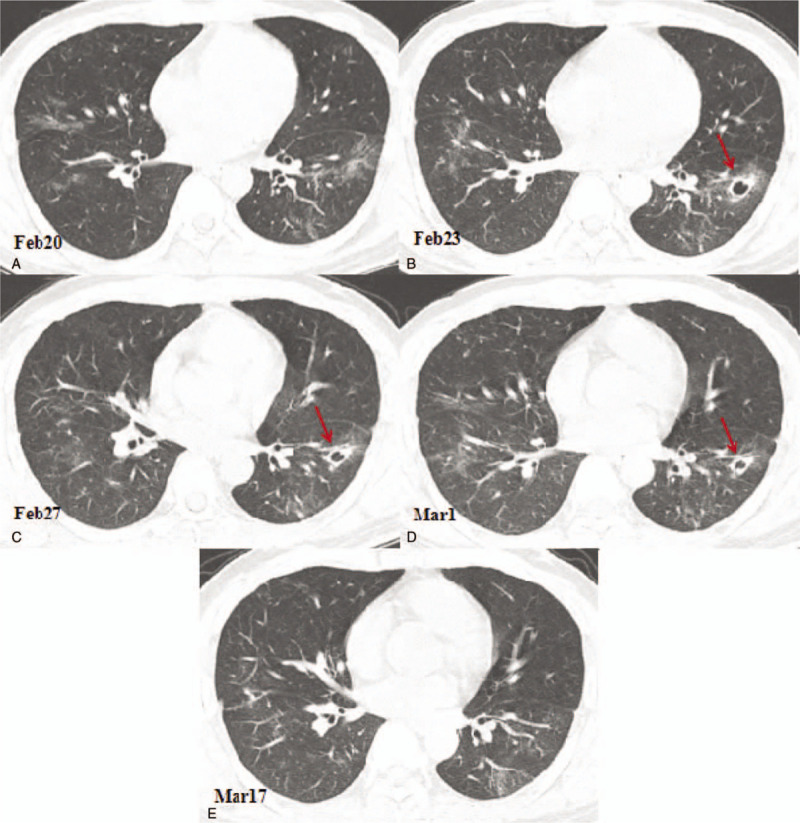
Chest computed tomography (CT) images showing cavitary changes in the inner anterior basal segment of the left lower lobe. (A) A CT image obtained on February 20, 2020 showing the ground glass opacities in the lungs. (B) Compared with the CT image obtained on February 20, 2020, the chest CT image of February 23, 2020 showing a new cavity with a partition in the anterior basal segment of the left lower lobe with a size of 15.0 × 13.0 mm (arrow). (C) A chest CT image showing that the anterior basal cavity in the left lower lobe is slightly reduced, with a size of 12.0 × 11.0 mm (arrow). (D) A chest CT image obtanined on March 1^st^ showing that the anterior basal cavity in the lower left lobe is further reduced, the size is 11.0 × 10.0 mm, and liquid-gas level appears (arrow). (E) A chest CT image obtained on March 17^th^ showing the cavity in the inner anterior basal segment of the left lower lobe was disappeared.

**Figure 3:**
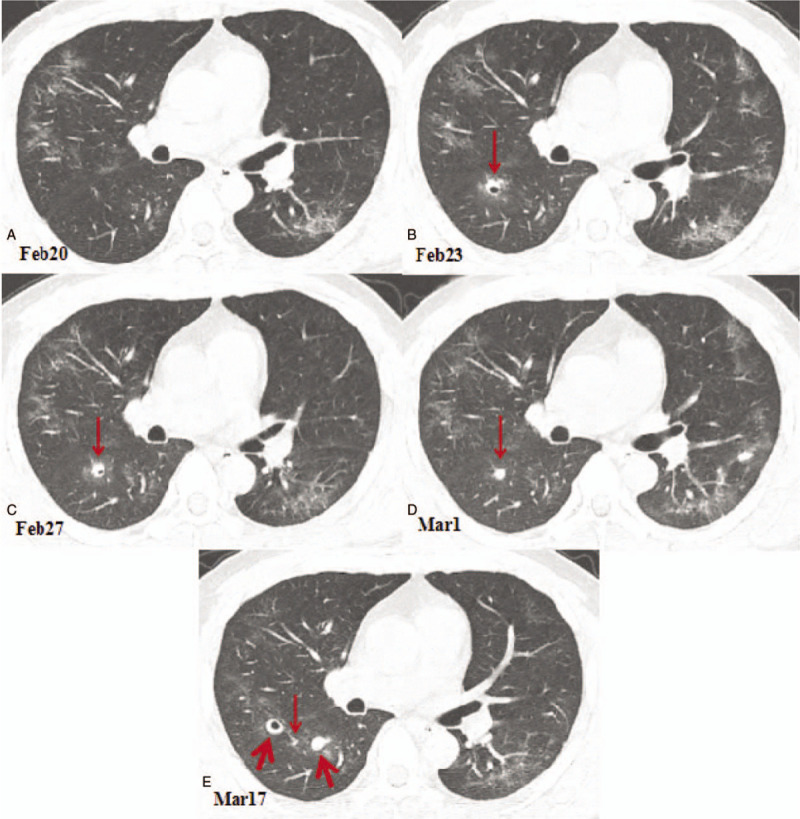
Chest computed tomography (CT) images showing cavitary change in the dorsal segment of the right lower lobe. (A) A CT image obtained on February 20, 2020 showing the ground glass opacities in the lungs. (B) Compared with the CT image obtained on February 20, 2020, the chest CT image obtained on February 23, 2020 showing a new cavity in the dorsal segment of the right lower lobe with a size of 8.2 × 7.1 mm (arrow). (C). A chest CT image obtained on February 27th showing a part of cavity in the dorsal segment of the right lower lobe was condensed, with a size of 7.7 × 6.3 mm (arrow). (D).A chest CT image obtained on March 1^st^ showing that the cavity of the lower right lobe was completely consolidated and shrunk to a size of 6.1 × 5.0 mm (arrow). (E) A chest CT image obtained on March 17^th^ showing that the lesion in the dorsal segment of the right lower lobe was almost completely absorbed, leaving only a punctate shadow (thin arrow). On the left side of the lesion, a new solid nodule with a size of 9.8 × 8.5 mm appears at the dorsal lobe of the right lower lobe (thick arrow); on the right side of the lesion, a new cavity appears in the posterior segment of the right upper lobe with a size of 11.5 × 10.3 mm (thick arrow).

After isolation in hospital, the patient received antiviral therapy consisting of 5 million units of α-interferon dissolved in 2-mL sterile injection water administered using atomization inhalation twice daily for 10 days and 500mg of ribavirin given intravenously twice daily for 10 days. His symptoms were relieved and his temperature returned to normal. The laboratory results also were returning to normal levels. On February 13, 2020, a blood test showed the white blood cell count had decreased to 7.11 × 10^9^ cells/L, the lymphocyte count to 0.7 × 10^9^ cells/L, the CRP level to 19.10 U/mL, and the ESR to 70.5 mm/h. On February 20, a chest CT scan showed that the ground glass opacities area had narrowed and the density of the lesions had decreased. However, a small cavity had appeared in the anterior segment of the left upper lobe with a size of 9.3 × 6.7 mm (including the walls, with a wall thickness of about 1–3 mm and a smooth inner wall; Fig. 1b). On February 23, a chest CT scan was performed again and we found that the area of ground glass opacities was further narrowed. The small cavity in the anterior segment of the left upper lobe (LUL) had consolidated, with a CT number of 17 Hounsfield units (HU) and a slightly reduced size of 7.8 × 6.3 mm, as shown in Figure 1C. However, a new cavity with a partition had appeared in the inner anterior basal segment of the left lower lobe (LLL) with a size of 15.0 × 13.0 mm and a wall thickness of about 2 mm (Fig. 2 A and B), and another new cavity had appeared in the dorsal segment of the right lower lobe (RLL) with a size of 8.2 × 7.1 mm (Fig. 3 A and B). The laboratory results on February 25, 2020 showed that the white blood cell count, the lymphocyte count, the CRP level, and the ESR had returned to normal levels. A CT scan on February 27 showed that the previous cavity in the anterior segment of the LUL was still consolidated with a size of 6.5 × 5.5 mm (Fig. 1D). The size of the cavity in the inner anterior basal segment of the LLL had decreased slightly to a size of 12.0 × 11.0 mm (Fig. 2C). A part of the cavity in the dorsal segment of the RLL had condensed to a size of 7.7 × 6.3 mm (Fig. 3C). The laboratory tests on February 28, 2020 returned to normal levels, with a white blood cell count of 5.57 × 10^9^ cells/L, a lymphocyte count of 1.63 × 10^9^ cells/L, a CRP level of 2.07 U/mL, and an ESR of 18 mm/h. After 2 consecutive negative COVID-19 nucleic acid tests on February 28 and 29, and a CT scan on March 1 revealing that the exudation had apparently been absorbed, the patient was discharged from the hospital and sent to a government designated hotel for quarantine observation.

**Figure 4:**
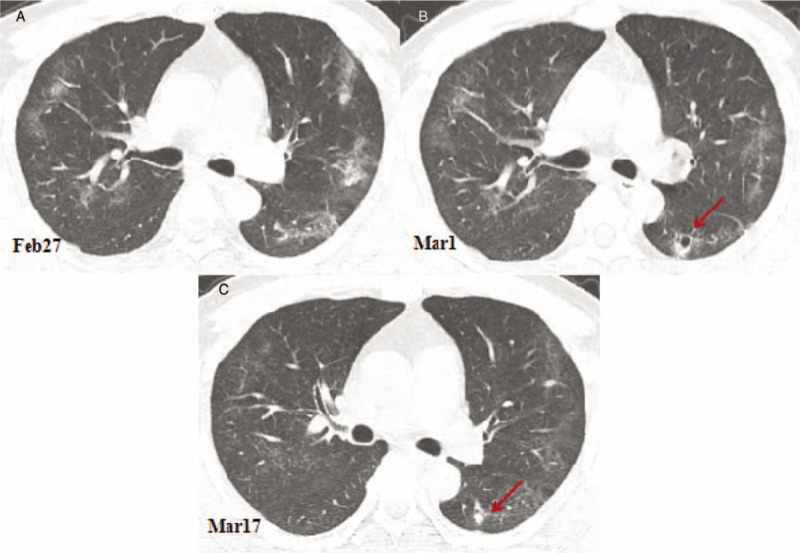
Computed tomography (CT) images showing the changes of a cavity in the dorsal segment of the right lower lobe. (A) A CT image obtained on February 27, 2020 showing the ground glass opacities in the lungs. (B) Compared with the CT image obtained on February 27, 2020, the chest CT image obtained on March 1st showing that the ground-glass opacities of both lungs were clearly absorbed, leaving a few linear opacities. A new cavity appears in the dorsal segment of the left lower lobe with a size of 10.1 × 9.7 mm (arrow). (C). Compare with the chest CT image obtained on March 1st, the chest CT image obtained on March 17th showing that ground glass opacities and linear opacities bilaterally were almost completely absorbed, and the cavity of the dorsal segment of the right lower lobe is solid and reduced in size 6.1 × 5.7 mm (arrow).

The CT scan on March 1 showed that the ground glass opacities in both lungs had clearly been absorbed, leaving a few linear opacities. The first detected cavity in the anterior segment of the LUL still looked like a solid nodule and had been reduced further to 5.0 × 4.5 mm (Fig. 1E). The second detected cavity in the inner anterior basal segment of the LLL had been reduced to 11.0 × 10.0 mm, and an air-fluid level was observed in the cavity (Fig. 2D). The third detected cavity in the dorsal segment of the RLL had consolidated completely, with its size reduced to 6.1 × 5.0 mm and a CT number of 19 HU (Fig. 3D). However, a new small thin-wall cavity had appeared in the dorsal segment of the LLL, with a size of 10.1 × 9.7 mm (Fig. 4A and B).

On March 17, 2020, the patient returned to the hospital for a recheck after 2 weeks of isolation observation. The blood test showed a white blood cell count of 5.37 × 10^9^ cells/L and a lymphocyte count of 1.63 × 10^9^ cells/L. A chest CT scan showed that the bilateral lung ground glass opacities had almost completely disappeared. The first detected cavity in the anterior segment of the LUL had decreased in size significantly, with a size of 2.5 × 1.5 mm (Fig. 1F). The second detected cavity in the inner anterior basal segment of the LLL had disappeared (Fig. 2E). The third detected cavity in the dorsal segment of the RLL had almost been absorbed completely, with only a punctiform shadow remaining. However, a new solid nodule with a size of 9.8 × 8.5 mm had appeared on the left side of the third cavity in the dorsal lobe of the lower lobe of the right lung, and a new cavity had appeared in the posterior segment of the upper lobe of the right lung with a size of 11.5 × 10.3 mm (Fig. 3E). The cavity in the dorsal segment of the LLL had consolidated and the size had decreased to 6.1 × 5.7 mm (Fig. 4B and C). The patient also underwent serological examination for SARS-CoV-2 antibody; the titer of SARS-CoV-2 antibody detected by chemiluminescence imunoassay was 80.45 (normal value range: 0–1 cut off index), indicating recovery. Based on these findings, a further 2 weeks of quarantine was recommended.

## 3 Discussion

COVID-19 is a pulmonary inflammatory disease. Based on the epidemiological evidence, the incubation period of the disease is 1 to 14 days, most often ranging from 3 to 7 days.^[5]^ The novel coronavirus nucleic acid can be detected in nasopharyngeal swabs, sputum, lower respiratory tract secretions, and feces in most of the patients. In the early stage of the disease, the number of peripheral white blood cells was normal or lower than normal, and the lymphocyte count was often lower than normal. Most patients had elevated CRP levels and a higher-than-normal ESR. After isolation for treatment, the majority of patients with COVID-19 pneumonia had their symptoms relieved and the laboratory test results returned to normal. But the prognosis for the elderly patients and those with chronic underlying diseases was poor.^[6]^ In our case, the epidemiological history, initial symptoms, laboratory results, and CT scan results were consistent with the diagnosis criteria for COVID-19 pneumonia and he was classified as moderate type.^[7]^ After he was hospitalized and isolated for treatment for 19 days, the clinical symptoms had been completely resolved, the CT scan showed that the acute inflammation had subsided significantly, and 2 consecutive novel coronavirus nucleic acid tests by rRT-PCR at 24-hour intervals had returned negative. He was then discharged from the hospital. After another 2 weeks of isolation in a government designated hotel, a serological examination showed high levels of SARS-CoV-2 antibody, indicating recovery.

A CT scan of the chest is particularly important in the diagnosis and evaluation of the treatment efficacy of patients with COVID-19 pneumonia. The typical early manifestations of COVID-19 pneumonia on a CT scan are multiple ground glass opacities in both lungs. With the development of the disease, the lesions could further expand and increase with consolidation. After effective isolation treatment, CT scans showed that the scope of the lesions had been reduced, the density had gradually decreased, the number of lesions had decreased, and the ground glass opacities could be absorbed. Some lesions could be transformed into linear opacities in a short period. However, there has not been any literature reporting small cavities in the lungs during the absorption phase.^[8,9]^ In this case, the initial findings on the CT scan were multiple ground glass opacities and linear opacities distributed in the peripheral third of the lungs, which are the typical pulmonary imaging manifestations of COVID-19 pneumonia. The CT scans were repeated after the patient was hospitalized, showing that the ground glass opacities had been reduced by absorption and few linear opacities remained. The unique manifestation in this case was the small cavities in both lungs during the absorption phase. These small cavities developed into consolidated nodules with clear edges and gradually shrank or disappeared.

A pathological examination of COVID-19 pneumonia revealed the formation of serous, fibrinous exudate and a transparent membrane in the alveolar cavity; congestion and edema of the blood vessels of the alveolar septum; the focal hemorrhage, necrosis, and hemorrhagic infarction of the lung tissue; alveolar infiltration and pulmonary interstitial fibrosis; a cavity in the epithelium of the bronchial mucosa in the lungs, and the formation of mucus and mucus plugs in the cavity; and a small number of over-inflated alveoli, alveolar septal rupture or cystic cavity formation.^[7,10]^ The small cavities in the lungs of this patient during the absorption period might be explained by the focal hemorrhage and necrosis of the lung tissue forming these small cavities after drainage through bronchioles; the congested blood vessels of the alveolar septum around the cavity and the edema around the cavity formed the thin wall. The alveoli around the cavity might have produced the serous fluid, fibrinous substances, and so on, that then filled the cavity, so the small cavities gradually consolidated. When the inflammation had been controlled and the exudation had subsided, the nodule shrank or was absorbed. The small cavities might also be explained by the fact that the bronchioles in the lungs were blocked by mucus and mucus plugs, the over-inflation of the alveoli, the rupturing of the alveolar septum, and the subsequent formation of small cysts, which appear as small cavities on the chest CT. The cavity consolidated and then shrank, eventually being absorbed or disappearing. In this case, we found new cavities 15 days after he was discharged from hospital for quarantine in a government designated hotel. Although 2 consecutive nucleic acid tests at two 24-hour apart returned negative results and the titer of the antibody indicating recovery, another 14-day quarantine was suggested because of the possibility of low-level viruses in the lungs.

In summary, although rRT-PCR assay remains the golden standard for the diagnosis of COVID-19, the high false negative rate limits the prompt diagnosis of this disease.^[11–13]^ Therefore, the CT scan plays a key role in diagnosing and fighting this infectious disease. The imaging changes in COVID-19 pneumonia are diverse, and a chest CT is important in the management of COVID-19. The small cavities in the lungs on a chest CT may be a manifestation of COVID-19 in the recovery period. Although the two consecutive nucleic acid tests at two 24-hour apart were negative for this patient, the possibility of low-level viruses in the lungs warrants quarantine observation and further evaluation.

## Author contributions

**Conceptualization:** Song Peng, Jinqing Chen.

**Data curation:** Jinqing Chen, Bangjun Zhang.

**Investigation:** Zhifeng Liu, Lang Liu.

**Methodology:** Song Peng, Zhifeng Liu, Lang Liu.

**Project administration:** Jinqing Chen, Bangjun Zhang.

**Resources:** Jinqing Chen,Song Peng.

**Supervision:** Wendy Zhang.

**Writing–original draft:** Song Peng, Jinqing Chen.

**Writing–review & editing:** Song Peng, Wendy Zhang.
